# Non-coding RNAs in hepatocellular carcinoma: Insights into regulatory mechanisms, clinical significance, and therapeutic potential

**DOI:** 10.3389/fimmu.2022.985815

**Published:** 2022-10-10

**Authors:** Qin Han, Mengchen Wang, Xi Dong, Fei Wei, Yun Luo, Xiaobo Sun

**Affiliations:** ^1^ Institute of Medicinal Plant Development, Peking Union Medical College and Chinese Academy of Medical Sciences, Beijing, China; ^2^ Key Laboratory of Bioactive Substances and Resources Utilization of Chinese Herbal Medicine, Ministry of Education, Institute of Medicinal Plant Development, Chinese Academy of Medical Sciences and Peking Union Medical College, Beijing, China; ^3^ Beijing Key Laboratory of Innovative Drug Discovery of Traditional Chinese Medicine (Natural Medicine) and Translational Medicine, Institute of Medicinal Plant Development, Peking Union Medical College and Chinese Academy of Medical Sciences, Beijing, China; ^4^ Key Laboratory for Research and Evaluation of Pharmacovigilance, Institute of Medicinal Plant Development, Peking Union Medical College and Chinese Academy of Medical Sciences, Beijing, China

**Keywords:** hepatocellular carcinoma, ncRNA, mechanism, biomarker, small-molecule modulator

## Abstract

Hepatocellular carcinoma (HCC) is a complex and heterogeneous malignancy with high incidence and poor prognosis. In addition, owing to the lack of diagnostic and prognostic markers, current multimodal treatment options fail to achieve satisfactory outcomes. Tumor immune microenvironment (TIME), angiogenesis, epithelial-mesenchymal transition (EMT), invasion, metastasis, metabolism, and drug resistance are important factors influencing tumor development and therapy. The intercellular communication of these important processes is mediated by a variety of bioactive molecules to regulate pathophysiological processes in recipient cells. Among these bioactive molecules, non-coding RNAs (ncRNAs), including microRNAs (miRNAs), long non-coding RNAs (lncRNAs), and circular RNAs (circRNAs), account for a large part of the human transcriptome, and their dysregulation affects the progression of HCC. The purpose of this review is to evaluate the potential regulatory mechanisms of ncRNAs in HCC, summarize novel biomarkers from somatic fluids (plasma/serum/urine), and explore the potential of some small-molecule modulators as drugs. Thus, through this review, we aim to contribute to a deeper understanding of the regulatory mechanisms, early diagnosis, prognosis, and precise treatment of HCC.

## Introduction

Hepatocellular carcinoma (HCC) is the sixth most common malignancy and the third leading cause of cancer-related deaths globally, posing a serious threat to human health and life (1, 2). Until now, mainstay curative treatments have included surgical resection, liver transplantation, radiofrequency ablation (RFA), transarterial chemoembolization (TACE), transarterial embolization (TAE), and systemic treatment with molecular-targeted agents. Despite great breakthroughs, current treatments have failed to deliver satisfactory outcomes, with an overall 5-year survival rate of only 12% (3–5). This failure is attributed to high heterogeneity, frequent recurrence, and drug resistance of HCC (6, 7). In addition, owing to the lack of reliable biomarkers, most patients progress to the intermediate and advanced stages of HCC at the time of diagnosis, regrettably missing the optimal treatment window. Therefore, deepening the understanding of the molecular mechanism to develop new therapeutic strategies and identifying biomarkers that can be effectively monitored at an early-stage is crucial in the fight against HCC.

The development of HCC is a multifactorial process. In recent years, immunotherapy has attracted extensive attention and has emerged as the next-generation therapy since the approval of immune checkpoint inhibitors (ICIs). However, studies have shown that its efficacy is closely related to the state of the tumor immune microenvironment (TIME). Essentially, anti-tumor immune efficacy mainly depends on the status and function of the immune cells in the TIME. Thus, it is necessary to elucidate the immune microenvironment of HCC to select appropriate ICIs (7). Angiogenesis not only delivers oxygen and nutrients to the growing tumor but also transports tumor cells to the metastatic site (8). Epithelial-mesenchymal transition (EMT) is a complex phenotypic event that directly affects changes in the characteristic features of HCC, such as occurrence, migration, invasion, metastasis, and even drug resistance (9, 10). Following primary tumor growth, angiogenesis and EMT, tumors are more prone to invasion and metastasis, which plays a key role in limiting patient outcomes overall. Therefore, there is an urgent need to explore invasive-metastatic cascade response of HCC (11, 12). Dysregulation of tumor cell metabolic activity may impair anti-tumor response, whereas metabolic reprogramming secures energy and substrates for the tumor (13, 14). Even more disastrous is drug resistance to chemotherapeutic agents in HCC. According to reports, mortality due to drug resistance accounts for more than 90% of cancer-specific mortality (15). In short, the above-mentioned issues are key barriers to the successful treatment of HCC.

Non-coding RNAs (ncRNAs) are endogenous RNAs accounting for the majority (98%) of the transcribed genome. They were once regarded as “dark matter” because of their lack of ability to encode proteins. After years of exploration, they have been found to act as important signaling molecules in the regulation of key cellular pathways (16–18). They are abundant and stable and mainly include microRNAs (miRNAs), long non-coding RNAs (lncRNAs), and circular RNAs (circRNAs). Approximately 30% of genes in the human body are regulated by miRNAs, which are the most abundant and studied group of ncRNAs (19). miRNAs regulate gene expression by binding to DNA, RNA, or proteins, which further regulates various biological functions (20). LncRNAs are linear RNAs with a transcript length of > 200 nucleotides, they have more diverse modes of action than miRNAs as the roles of spatial and temporal lncRNAs in cell physiology and pathology have gradually become clear. They can act as signals, decoys, scaffolds, or guides. Even the same kind of lncRNAs can function *via* different mechanisms (21–23). Emerging evidence indicates that circRNAs are novel ncRNAs that are related to many pathological diseases. In contrast to the standard splicing of linear RNAs, circRNAs are closed-loop structures produced by back-splicing (24). CircRNAs exhibit high abundance, diversity, sequence conservation among species, stability, tissue specificity, and tumor stage-dependent characteristics (25, 26). They exert their functions by binding to RNA-binding proteins, sponging miRNAs, translating into peptides or proteins, regulating gene transcription, and competing with canonical splicing (26, 27). To date, studies have found that these functional molecules mediate intercellular communication, which plays a non-negligible role in the TIME, angiogenesis, EMT, invasion, metastasis, metabolism, and drug resistance (14, 28–30). ncRNAs can also be detected as circulating molecules in the serum/plasma/urine, indicating that they are of great significance in early diagnosis and prognosis. In addition, small-molecule modulators that target ncRNAs are of great use. Hence, ncRNAs hold great promise as potential biomarkers or therapeutic targets (31). Herein, we summarize the latest findings on ncRNAs (miRNAs, lncRNAs, and circRNAs) that affect various aspects of HCC, including TIME, angiogenesis, EMT, invasion, metastasis, metabolism, and drug resistance. The potential uses of ncRNAs in cancer diagnosis/prognosis and the therapeutic activity of small-molecule modulators that selectively target ncRNAs are also summarized.

## Regulatory mechanisms of ncRNAs in HCC

### Regulation of TIME

**Figure 1:**
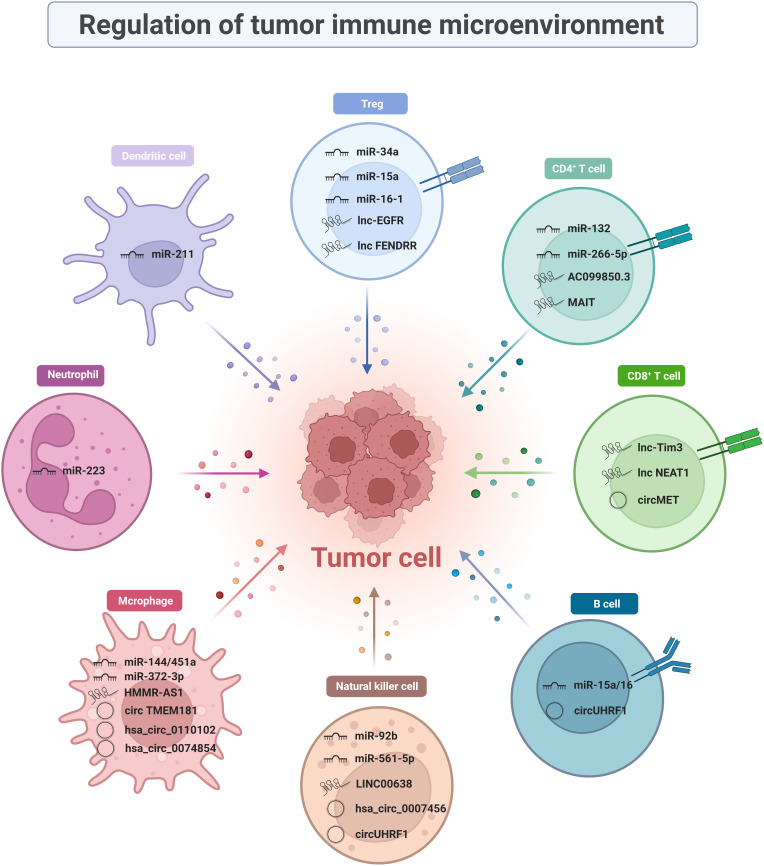
Regulation of ncRNAs to tumor immune microenvironment.

Immune cells are complex, heterogeneous cells with different developmental stages and functional subpopulations. ncRNAs present in the TIME can regulate immune cells and influence the development of tumor immune responses. The molecular mechanisms by which ncRNAs regulate immune cellular subpopulations in TIME and tumor immune response development are described in detail in this section (**Figure 1**).

#### T cells

##### CD8^+^ T cells

CD8^+^ T cells are key players that perform anti-tumor immune functions in the TIME and characteristic markers of good prognosis in HCC. They mediate target cell apoptosis by secreting perforin and granzyme or by expressing Fas ligand (FasL) (32, 33). Single-cell RNA sequencing indicated that the effector CD8^+^ T cells in advanced HCC patients were depleted and weakened in cytotoxicity compared to those in early-stage of HCC, which may lead to impaired anti-tumor function (34). Unfortunately, high expression of activation/depletion markers (notably PD1, TIM3, and LAG3) on the surface of CD8^+^ T cells shifts high-density cytotoxic T cells towards immune exclusion (35). This phenomenon raises a prerequisite condition for the application of immune checkpoints, that is, how to convert “cold tumors” into “hot tumors”, which is also a pressing issue to be addressed. Some studies have found that targeting ncRNAs can alter CD8^+^ T cell activity and restore anti-tumor immune function in the tumor microenvironment (TME). Since then, considerable research has been conducted that ncRNAs regulate the anti-tumor effects of CD8^+^ T cells.

Currently, multiple lncRNA biomarkers obtained by invasive procedures show a great capability in mediating the interaction between tumor cells and CD8^+^ T cells, which has generated great research interest. Tim-3 is a negatively regulated T-cell-dependent immune responses’ immune checkpoint that serves as a perfect target for next-generation immunotherapy owing to its precision and specificity. A previous study reported that Lnc-Tim3, which is highly expressed in tumor-infiltrating CD8^+^ T cells, specifically bond to Tim-3 and blocked the interaction with Bat3. This phenomenon inhibits downstream Lck/NFAT1/AP-1 signal transduction, thereby exacerbating CD8^+^ T lymphocyte exhaustion (36). Other clinical studies found that the expression of NEAT1 was upregulated in peripheral blood mononuclear cells (PBMCs) from patients with HCC and could interfere with Tim-3 expression by binding to miR-155. Downregulation of NEAT1 inhibits apoptosis in CD8^+^ T cell and enhances cytolytic activity, thereby inhibiting tumor growth (37).

CircMET is a widely studied circRNA that is aberrantly expressed in HCC tumors (38). Mice subcutaneously implanted with Hep1-6-circMET had a smaller tumor burden and a higher density of tumor-infiltrating CD8^+^ T cells than those implanted with Hep1-6-control cell lines. This indicates that circMET is detrimental to CD8^+^ T cell infiltration, and in-depth studies revealed that it achieved its goal through the miR-30-5p/Snail/dipeptidyl peptidase-4 (DPP4) axis. Based on the combined clinical approach of DPP4 inhibitor and anti-PD1 blocking immunotherapy, it further validated that the DPP4 inhibitor sitagliptin can enhance CD8^+^ T cell trafficking and increase infiltration levels, thereby improving the efficacy of PD1 blockade immunotherapy, supporting the clinical application of combining DPP4 inhibitors with anti-PD1 blocking immunotherapy.

##### CD4^+^ T cells

CD4^+^ T cells should not be underestimated because they perform multiple functions in the adaptive immune system. They are not only able to kill tumor cells directly (cytotoxic CD4^+^ T cells) but are also well-known for their indirect role in the TIME as T helper (Th) cells. They can coordinate the enhancement of other anti-tumor effector cell functions, such as CD8^+^ T cell function and macrophage phagocytosis. Differentiation into various subpopulations, such as Th1, Th2, Th17, and regulatory T (Treg) cells, is induced in different cytokine environments, with different on anti-tumor effects (39, 40). Since the study of Treg cells requires an in-depth review of available information, it was singled out for discussion. ncRNAs are an integral part of gene expression networks, which dynamically regulate CD4^+^ T cells’ differentiation and plasticity. Dysregulation of certain ncRNAs in cancer cells can increase the levels of immunosuppressive factors, which in turn contributes to immune privilege (41).

Interestingly, lncRNA AC099850.3 exerts oncogenic effects *via* the PRR11/PI3K/Akt signaling pathway. An immune infiltration analysis revealed that T follicular helper cells and CD4^+^ memory T cells were activated while CD8^+^ T cells and monocytes were suppressed when AC099850.3 was up-regulated, explaining the oncogenicity of AC099850.3 (42). LncRNA MAIT is mainly expressed in CD4^+^ T cells from HCC tumor tissues and paracancerous tissues. It is not only positively correlated with the level of CD4^+^ T cell infiltration, but also with immunosuppressive molecules, such as PD-1, PD-L1, and CTLA4 (43). miR-26b-5p targeting proviral integrations of moloney virus 2 (PIM2) can affect the secretion of tumor necrosis factor α (TNF-α), interferon-γ (IFN-γ), interleukin-6 (IL-6), and interleukin-2 (IL-2) in CD4^+^ T cells (44). Th17 cells mediate pro-inflammatory functions by secreting cytokines (such as IL-17, IL-21, and L-22), and they participate in many organ-specific autoimmune diseases. Furthermore, ncRNA functions are being actively explored in Th17 cells in the TIME. miR-132 expression is much higher in CD4 IL-17^+^ cells than in CD4 IL-17^-^ cells. miR-132 mediates Th17 cell differentiation by promoting IL-22 expression, which in turn enhances hepatic stellate cell (HSC) activation and induces tumor migration (45).

##### Regulatory T cells (tregs)

Tregs, a regulatory subpopulation of infiltrating CD4^+^ T cells, are recognized as a major suppressive component of the immune system. They are extremely important for the formation of an immunosuppressive microenvironment in HCC (46, 47). Tregs are critical in maintaining self-tolerance and immune homeostasis and are co-opted by tumor cells to evade immune surveillance. They are up-regulated in tumor tissues and peripheral blood from patients with HCC or mice than in healthy individuals (48). High FOXP3 expression on the cell surface is a distinctive feature of these cells (47, 49). Several studies have reported that the biological behavior and function of Tregs are partially dependent on the regulation of ncRNAs. ncRNAs affect the expression of immune-related cytokines and growth factors (e.g., IL-2) by regulating the secretion of chemokines (e.g., CCL22), which further affects the function and differentiation of Tregs that accumulate in the TIME (50, 51). Several studies have provided evidence suggesting that miR-34a, miR-15a, miR-16-1, lnc-EGFR, and lncRNA FENDRR play a crucial role in affecting Tregs in the HCC microenvironment.

CCL22 is a chemokine required for Tregs to exceed CD8+ T cells. As early as 2012, miR-34a strongly supported the idea that ncRNAs affect the immunosuppressive function of TIME by regulating the secretion of CCL22. Yang et al. found that elevated activity of tumor growth factor-β (TGF-β) suppressed miR-34a expression and dose-dependently enhanced production of CCL22 in PVTT-1 cells. This blocks the strong binding of CCL2 to CCR4 on the surface of Tregs, resulting in attenuated Treg cell recruitment and immune escape suppression (52). Similarly, miR-15a and miR-16-1 directly target NF-κB to impair CCL22 transcription. Subsequently, activated NF-κB/CCL22 signaling attenuates the hepatic recruitment of Tregs. Such biological activity also upregulates CD80 expression in Kupfer cells (KCs) and CD28 in Tregs, facilitating communication between KCs and Tregs (53). A study confirmed the existence of a forward-feedback loop lnc-EGFR-EGFR-NF-AT1/AP1-lnc-EGFR in Tregs as a facilitating mechanism for HCC (54). In addition, loss of GADD45B can upregulate the number of Tregs. However, the tumor suppressor lncRNA FENDRR targets GADD45B as a miR-423-5p sponge to suppress the secretion of immune-related factors TGF-β, vascular endothelial growth factor (VEGF), IL-2, and IL-10, thereby suppressing Treg-mediated immune escape (55).

#### B cells

B lymphocytes are derived from pluripotent stem cells in the bone marrow. They play a dual role in tumor immunity by supporting or suppressing anti-tumor immunity (56). B cells may act as antigen-presenting cells to enhance humoral and cellular responses to tumors. However, the strong prognosis of tumor-infiltrating B cells (TIL-Bs) in cancer reject this idea. Therefore, it is difficult to determine the specific role of B cells (57). One claim is that miRNAs may influence the differentiation of the regulatory B cells (Bregs). Similar to Tregs, Bregs produce high levels of IL-10 and suppress the host immune response, thereby exerting a pro-tumor effect (58). An increased frequency of CD19^+^ Tim-1 cells and tumor growth have been observed in young miR-15a/16^-/-^ mice transplanted with HCC cells. This phenomenon depends on the efficiency of microRNA cluster of miR-15a/16 that enhances STAT3 activity. Then STAT3 activation contributes to IL-10 production by CD19 Tim-1 cells, and finally promotes Bregs’ activity (59). Pseudogenes are a special type of lncRNAs. The expression of pseudogenes RP11-424C20.2 correlates with the level of tumor-infiltrating immunocytes, including B cells (60). It linked high levels of B cells with worse outcomes for thymoma (THYM) patients. Necessarily, further studies are needed to explore how other ncRNAs exert regulatory effects on B cells and their specific mechanisms.

#### Natural killer cells

NK cells can exert their effects as an essential component of innate immunity even without prior stimulation, which constitutes the first line of host immunological defense against cancer cell invasion. NK cells lead to target cell apoptosis by secreting perforin and granzyme, expressing FasL, and mediating antibody-dependent cellular cytotoxicity (ADCC) (61, 62). Changes in the phenotypes and functions of NK cells have been detected both in patients with aggressive human liver cancer and transgenic mouse models (63, 64). Single-cell RNA sequencing and flow cytometry of innate lymphoid cells (ILCs) revealed that NK cells lose their cytotoxic profile as they transition into NK-like-ILC3 cells (65). Several therapies of NK cell-mediated ADCC have been evaluated in clinical trials. Undoubtedly, NK cells are promising candidates for the development of advanced cancer immunotherapy. Evidence collected so far suggested that multiple ncRNAs mediate interactions between NK and HCC cells.

CD69 is an NK cell activation marker that mediates NK cell cytotoxicity. When transferred to NK cells, miR-92b causes CD69 downregulation and cytotoxic damage (66). Chen *et al.* found that miR-137, miR-149-5p, and miR-561-5p are associated with the innate immune response, especially miR-561-5p. Additional evidence has shown some differences in chemotaxis and function among different NK cell subsets. miR-561-5p attenuated the anti-tumor response by downregulating CX3CL1 messenger RNA (mRNA) to reduce the function of CX_3_CR1 NK cells (67).

Recent studies have found that the LINC00638 is mainly enriched in eight signaling pathways, in which NK cell-mediated cytotoxic pathway is highly correlated with immune infiltration. In HCC tumor tissues, overexpression of ULBP1 can recruit NK cells to the tumor and leads to immune escape when accompanied by PD-L1 expression. Mechanistically, LINC00638 can achieve this goal by acting as a sponge for miR-4732-3p and eliminating the inhibition of ULBP1 expression (68).

Notably, attention has gradually been focused on circRNAs in HCC immunity regulation. One of the most well-known examples is circUHRF1 (hsa_circ_0048677). Highly expressed circUHRF1 sponges miR-449c-5p to upregulate the expression of the downstream target gene, T-cell immunoglobulin mucin 3 (TIM-3), thereby reducing the secretion of TNF-α and IFN-γ, which ultimately promote the immune escape of HCC cells (69). Moreover, the downregulation of hsa_circ_0007456 reduces NK cell susceptibility and attenuates their binding by the downstream miR-6852-3p/ICAM-1 axis, thus promoting the immune escape of HCC cells (70).

#### Tumor-associated macrophages

Macrophages are key mediators of tissue homeostasis. They can directly kill tumor cells by phagocytizing massive pathogens (71). Additionally, they are vital antigen-presenting cells that activate endogenous anti-tumor T cell responses. They are highly plastic and can be classified into two subtypes: classical pro-inflammatory activation (M1-like macrophages) and alternative anti-inflammatory activation (M2-like macrophages) (72). The tumor recruits them into the TIME and induces the formation of TAMs, which are crucial for promoting the immunosuppressive microenvironment, tumor cell invasion, angiogenic switch, and immune escape of malignant cells.

Previous studies have shown roles of miRNAs in M1/M2 macrophage polarization, which mediates the onset and growth of HCC. Zhao *et al.* reported that the CpG island deletion (ΔCpG) of the miR-144/miR-451a promoter induces chromatin conformational remodeling. It increases the expression of miR-144/miR-451a while decreasing the expression of hepatocyte growth factor (HGF) and macrophage migration inhibitory factor (MIF), conferring the paracrine activation of macrophage M1-like repolarization (73).

Many studies have revealed the mechanism of action of lncRNAs in TAM polarization regulation. Overexpression of PART1 promotes macrophage M2-like polarization by affecting the miR-372-3p/TLR4 axis (74). Similarly, promoting competitive adsorption of miR-147a by lncRNA HMMR-AS1 in a hypoxic environment affected ARID3A-mediated macrophage polarization (75). In addition, it has been found that LINC00662, lncRNA TUC339, lncRNA MALAT1, PCED1B-AS1, lnc-Ma301, and lncRNA cox-2 can promote the differentiation of TAMs into the M2 phenotype and inhibit the anti-tumor response (76–81).

Other important studies have confirmed the crucial role of circRNAs in orchestrating macrophage polarization in HCC progression. The use of macrophage-specific CD39-knockout mice showed that circTMEM181 upregulates CD39 expression in macrophages and CD73 expression in HCC cells. The cooperation between CD39 and CD73 triggers eATP-adenosine activation, thereby promoting immunosuppression (82). Hsa_circ_0110102 may act as a sponge for miR-580-5p to regulate target gene function. It regulates the secretion of CCL2 into the TME by decreasing PPARα expression. The release of pro-inflammatory cytokines from macrophages was inhibited by modulating the COX-2/PGE2 pathway. Thus, hsa_circ_0074854 may be a potential prognostic predictor or therapeutic target for HCC (83). Besides, the knockdown of hsa_circ_0074854 can inhibit M2 macrophage polarization both *in vitro* and *in vivo*. Mechanistically, the downregulation of hsa_circ_0074854 inhibits macrophage M2 polarization by interacting with human antigen R (HuR), thereby inhibiting the migration and invasion of HCC cells (84).

#### Other immune cells

In addition to the above-mentioned immune cells, ncRNAs exert regulatory effects on other immune cells, such as tumor-associated neutrophils (TANs) and dendritic cells (DCs). TAN is the most abundant circulating leukocyte in humans that mediates tumor growth and progression. It has two distinct phenotypes-N1 (anti-tumor) and N2 (pro-tumor), exhibiting functional heterogeneity in response to different stimuli (85). Therefore, it is a potent modulator of TIME. The deregulation of miR-223 may play a role in a range of liver diseases by affecting neutrophil infiltration. In addition, miR-223 expression positively correlates with the differentiation of granulocyte-monocyte progenitor cells into granulocytes (86). As antigen-presenting cells, DCs can induce primary immune responses (87). Wu pointed out that lncRNA ASB16-AS1 negatively correlated with dendritic cells and neutrophils as validated in five HCC cell lines (88). The mechanism by which ncRNAs regulate immune cells in the TIME is still in its infancy and needs to be supported by further studies.

### Regulation of tumor angiogenesis

In the tumor growth environment, there is a dynamic imbalance between pro- and anti-angiogenic factors. As a hypervascular tumor, HCC tends to induce the secretion of proangiogenic factors such as VEGF, angiopoietin-1 (ANGPT1), platelet-derived growth factor (PDGF), and basic fibroblast growth factor (bFGF). This phenomenon contributes to angiogenesis, enabling tumors to receive adequate nutritional support and expel metabolic waste and carbon dioxide, leading to continuous tumor growth and progression (8, 89). Overexpression of VEGF contributes to vascular network development, endothelial cell proliferation, and tube formation (90, 91). Current anti-angiogenesis drugs, such as bevacizumab and ramucirumab, mostly target the VEGF signaling pathways. Accumulating evidence suggests that ncRNAs regulate tumor progression by interacting with VEGF in HCC. A previous study showed that miR-195 is negatively correlated with angiogenesis which directly inhibits VEGF levels and VEGF receptor 2 signaling in endothelial cells (92). Another study showed that the lncRNA PAARH positively correlated with vascular invasion in HCC tissues, upregulated VEGF expression and microvascular density. PAARH facilitated the recruitment of HIF-1α to the VEGF promoter, which caused high VEGF expression (93). In addition to VEGF, ncRNAs interact with other growth factors. By targeting ANGPT1, miR-375 suppresses proangiogenic activity and miR-3682-3p weakens angiogenesis both affecting tumor progression (94, 95).

ncRNAs promote angiogenesis through crosstalk with cancer-associated endothelial cells (ECs), affecting tube formation (30). It has been shown that miR-210 targets SMAD4 and STAT6 to stimulate EC tubulogenesis *in vitro* and angiogenesis *in vivo*. STAT6 alleviates the inhibitory effects of IL-13 on human coronary artery EC migration and tube formation; however, the mechanism of action of SMAD4 on ECs remains unknown (96). In contrast, ncRNAs inhibit the permeability of ECs to mediate cancer cell proliferation, which are potential targets for anti-angiogenic therapies. For example, miR-638 can disrupt endothelial tight junctions and enhance the permeability of FITC-dextran by downregulating VE-cadherin and ZO-1 expression in non-cancerous regions of the liver (97). Another study has explained the specific mechanism by which circRNA-100,338 regulates endothelial permeability. CircRNA-100,338 affects cell permeability and vasculogenic mimicry (VM) capability by interacting with NOVA2 and inhibits HCC growth by binding to IFN-α (98).

**Figure 2:**
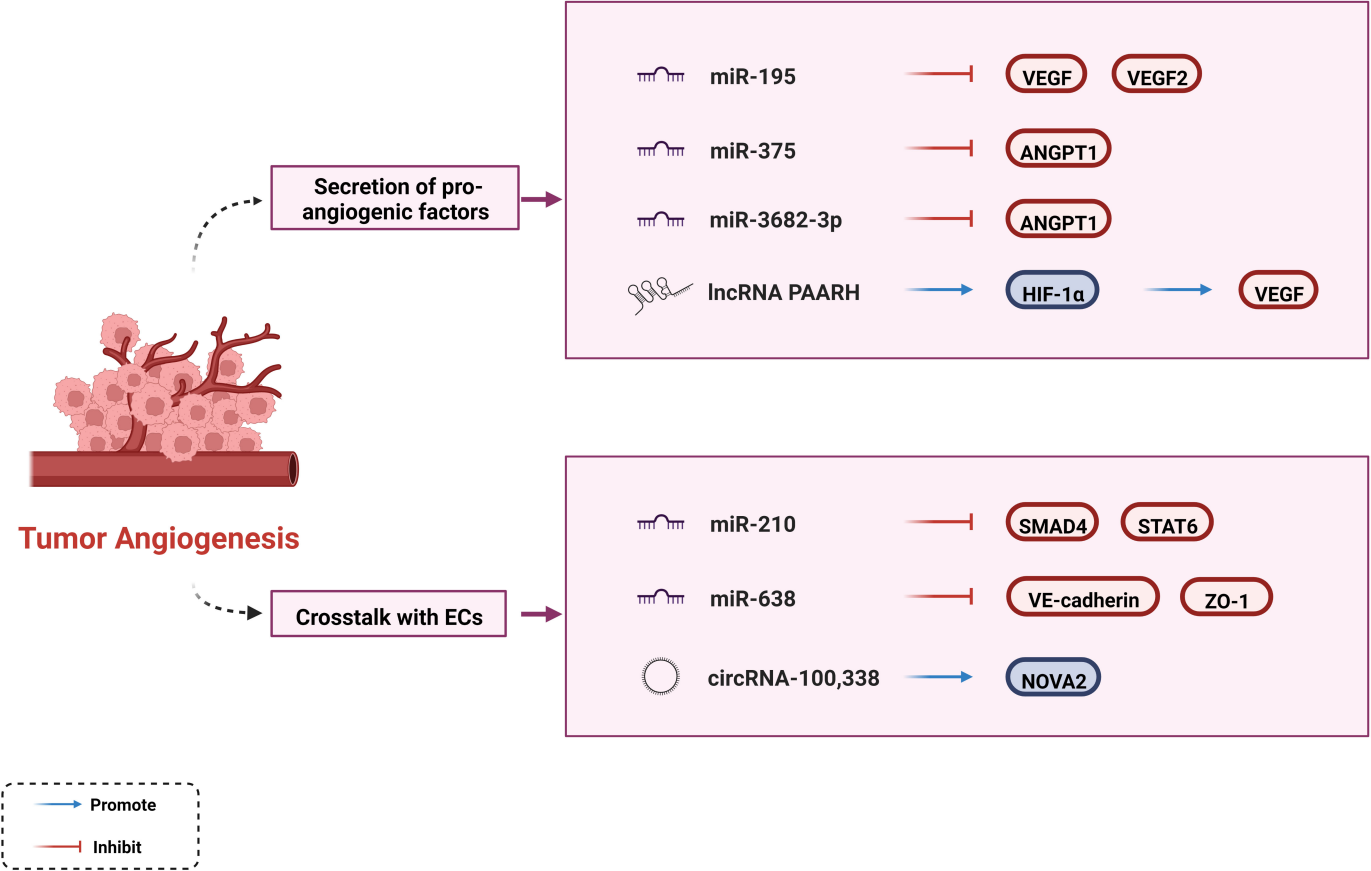
Regulatory mechanisms of ncRNAs to tumor angiogenesis.

Collectively, ncRNAs can regulate angiogenic activity through different pathways (**Figure 2**). ncRNAs may be emerging targets for anti-angiogenic therapies. The clinical benefits of multi-kinase inhibitors (such as Sorafenib and Lenvatinib) that target VEGF and its receptors have not been as good as initially expected, perhaps partly because of the off-target effect of anti-tumor agents (99). Synergistic biological effects exist between antiangiogenic agents and ICIs, and their toxicity profiles do not overlap (99). Based on these characteristics, some experts have focused on promoting the ncRNA-targeting combinations of ICI inhibitors with Sorafenib or other anti-angiogenic drugs, which could contribute through the aforementioned mechanism.

### Regulation of EMT

EMT is indispensable at all life stages from embryonic development to death, and it is essential for the acquisition of the invasive and metastatic characteristics of malignant tumors. Featured with reversibility, plasticity, and heterogeneity, EMT can alter multiple phenotypic changes including the loss of cell polarity, dissolution of intracellular junctions, and basement membrane detachment (9, 10). Epithelioid cell markers, such as β-catenin, E-cadherin, ZO-1, and claudin-1, were negatively correlated with the EMT process, while mesenchymal-like cell markers, such as Snail, twist, vimentin, N-cadherin, ZEB, and α-SMA, were positively correlated with the EMT process. Besides, certain signaling pathways facilitate the EMT process, such as the Wnt/β-catenin signaling pathway. An increasing number of studies have shown that ncRNAs act as mediators affecting the EMT process in tumor cells (28).

ZO-1 regulates cell material transport and maintains epithelial polarity. Circ-0004277 competitively binds to HuR and blocks the binding of ZO-1 and HuR, thereby stimulating EMT progression and promoting HCC (100). LncRNA TP53TG1 is a tumor suppressor gene that negatively correlates with N-calmodulin and vimentin, and positively correlates with E-cadherin and claudin-1 at the protein and RNA levels (101). Snail acts as a major player in EMT, whereas HOTAIR negatively regulates the EMT process (102). The results confirmed that HOTAIR-sbid, a HOTAIR deletion mutant, can impair the interaction between Snail and EZH2, thus affecting the capacity of Snail to convey EZH2 to specific epithelial target sites (i.e., HNF4α, E-calmodulin, and HNF1α). The design of such dominant-negative mutants opens new perspectives for highly specific future RNA therapeutics to counteract tumor progression (103). A recent investigation showed that the tumor-suppressive function of circPABPC1 is manifested by promoting the degradation of β1 integrin (ITGB1), a pivotal member of the integrin family, thereby reducing cell adhesion between cells and the extracellular matrix (104). In contrast, lncRNA TP53TG1 interacts with peroxiredoxin-4 (PRDX4) to promote its ubiquitin-mediated degradation, subsequently downregulating the levels of proteins involved in the Wnt/β-catenin pathway, thereby slowing down the EMT process (101). Notably, a growing body of information is available on different ncRNAs, such as miR-1246, lncRNA DLGAP1-AS1, and circMTO1, which regulate the Wnt/β-catenin pathway to affect the EMT process (105, 106). In addition, miR-92a-3p inhibits the PTEN/Akt/Snail pathway, miR-612 inhibits the Sp1/Nanog signaling pathway, and lncRNA LL22NC03-N14H11.1 activates the H-RAS/MAPK pathway to induce mitochondrial fission, all of which can explain the key role of ncRNAs in EMT progression (107–109).

**Figure 3:**
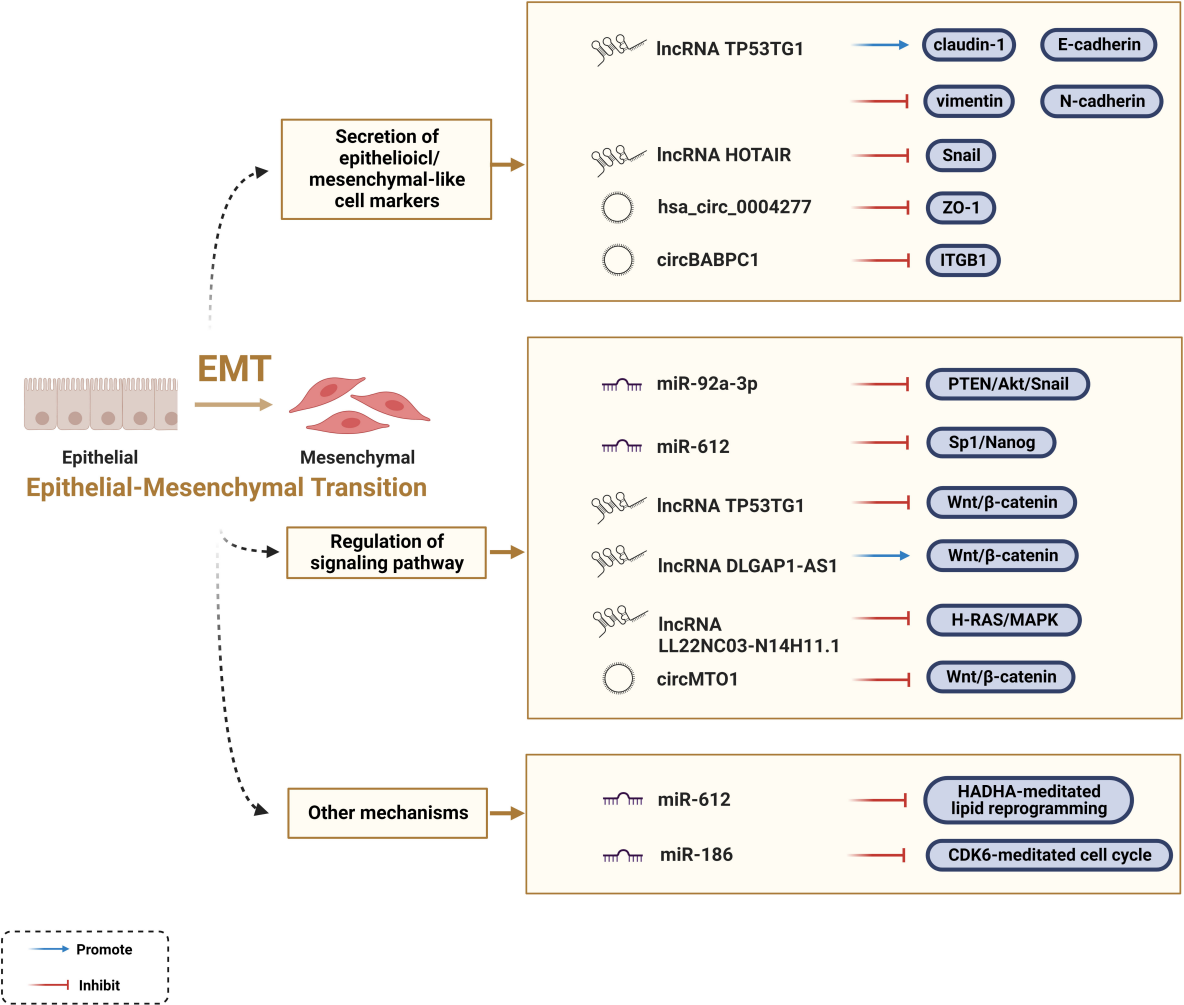
Regulatory mechanisms of ncRNAs to epithelial-mesenchymal transition.

Furthermore, scientists have elucidated how ncRNAs regulate the EMT process in HCC from some new perspectives. In addition to the Sp1/Nanog signaling pathway mentioned above, Liu *et al.* found that miR-612 regulates the EMT process through direct downstream target HADHA-mediated lipid reprogramming. Mechanistically, miR-612 affects invadopodia formation through HADHA-mediated alterations in the β-oxidation of fatty acids, cholesterol biosynthesis, and cell membrane fluidity (110). Evidence also indicates that cell cycle-related genes are involved in the regulation of ncRNAs in EMT; for example, miR-186 induces apoptosis by directly targeting cyclin-dependent kinase 6 (CDK6) (111). In conclusion, ncRNAs are key factors regulating the EMT process in HCC (**Figure 3**); however, their regulatory functions and underlying mechanisms remain to be elucidated.

### Regulation of tumor invasion and metastasis

Tumor invasion and metastasis are complex processes of multi-stage evolution and hallmarks of malignancy, and can lead to a low survival rate in patients with distal metastasis. Even more problematic is that the current clinical situation is not conducive for detecting dormant cancer cells and micro-metastasis. This further exacerbates the difficulty of treatment, which requires a deeper understanding of tumor invasive and metastatic mechanisms. The processes remodeling the extracellular microenvironment that ncRNA-induced, such as angiogenesis and EMT, are niches for tumor metastasis. Those have been described in detail in the previous two sections. Further, malignant cells’ ability to distant movement is a favorable niche for tumor metastasis (112). Research on ncRNAs in the complex invasive-metastatic cascade response has provided new insights into the molecular mechanisms involved in hepatocellular carcinoma.

Upregulation of miR-1251-5p in tissues of HCC patients is significantly associated with clinical stage, high tumor lymph node metastasis (TNM), and poor prognosis. miR-1251-5p overexpression promoted cell invasion *in vitro*, whereas miR-1251-5p silencing inhibited HCC cell invasion *in vivo*. A-kinase anchor protein 12 (AKAP12) exerts anti-tumor effects by acting as a scaffolding protein in signal transduction. The luciferase report showed that miR-1251-5p could directly target AKAP12 for oncogenic effects, and this mechanism was validated by AKAP12 knockdown rescuing the miR-1251-5p knockdown-attenuated cell invasion (113). miRNAs have been studied for a long time and there is now a wealth of data on their broad and critical role in tumor metastasis through target genes and signaling pathways, such as miR-195, miR-122, miR-103a-3p (92, 114, 115). LncRNAs regulate the expression of target genes by mediating miRNA activity. Metastasis-associated lung adenocarcinoma transcript 1 (MALAT1) is a widely studied lncRNA in cancer and is important for cancer-related pathway regulation. The luciferase reporter assay confirmed that MALAT1 negatively regulates miR-200a expression and is involved in the proliferation and invasion of Hep3B cells under hypoxia (116). It is well established that lncRNA is a key regulator of tumorigenesis. Liu’s research cascaded the exact correlation of this lncRNA to hepatocyte invasion and metastasis and revealing downstream regulatory pathways. The potential miRNA binding site of lncRNA BACE1-AS, miR-377-3p, was identified by a raw letter prediction and experimentally confirmed method. Further, the analysis showed that miR-377-3p negatively regulates CELF1, an RNA-binding protein that is a relative marker of malignancy (117). MMP9, a member of the zinc-dependent endopeptidase family, is a vital role in metastasis, especially in degrading ECM. Mouse xenograft models and mouse lung metastasis models confirmed the pro-tumor growth and lung metastasis role of circUBAP2. It negatively regulates HCC by acting as a competing endogenous RNAs(ceRNAs) for miR-194-3p and inhibiting MMP9 (118). In conclusion, complex biochemical and biological alterations in the tumor cells themselves and the associated stroma contribute to this aggressive phenotype. The involvement of ncRNAs in this process also expands the hope for finding new regulatory key nodes and therapeutic strategies for metastatic tumors.

### Regulation of tumor metabolism

Vasculature-restricted glucose and oxygen delivery is insufficient to supply uncontrolled proliferation of cancer cells. Therefore, cancer cells undergo metabolic reprogramming as a survival strategy. Cancer cells generate energy through aerobic glycolysis and lactic acid fermentation in a process rather than the TCA cycle to meet the energy demand of rapid cancer cell proliferation. This process is known as the Warburg effect. In this way, additional substrates and energy requirements can be provided for the biosynthesis of macromolecules (14, 119, 120). Several ncRNAs have been shown to rewire glycolytic networks (14). Zheng *et al.* found that LINC01554 negatively regulates the key rate-limiting enzyme pyruvate kinase isozyme M2 (PKM2) in the aerobic glycolysis pathway of HCC. However, whether this mechanism is due to ubiquitin-mediated degradation or as a scaffold remains to be further investigated. In contrast, LINC01554 inhibits the PI3K/Akt/mTOR signaling pathway, which is a central signaling pathway coordinating glucose uptake, glycogen synthesis, and tumorigenesis (121). Consistent with LINC01554, circRPN2 also facilitates glycolytic reprogramming through the Akt/mTOR pathway (122). In addition, it inhibits aerobic glycolysis by regulating the miR-183-5p/FOXO1 axis. Moreover, studies have verified that lncRNA TUG1, lncRNA RAET1K, and circFBLIM1 play a role in metabolic reprogramming by regulating glycolysis (123–125).

In addition to their roles in glycolytic metabolism, miR-21 and miR-122 play central roles in hepatic lipid metabolism and cholesterol synthesis relying on a complex lipogenic transcriptional network (126, 127). miR-21 regulates lipid metabolism through at least three pathophysiological pathways in a zebrafish model. One of the approaches is that miR-21 promotes hepatic lipid accumulation *via* the PTS (PI3K/Akt, TGF-β/SMAD3, STAT3) signaling networks. As for miR-122, free fatty acid (FFA) increases miR-122 secretion and transportation by activating retinoic acid-related orphan receptor alpha (ROR-α). In turn, miR-122 reduces the mRNAs levels of genes (*Agpat1* and *Dgat1*) involved in triglyceride synthesis.

**Figure 4:**
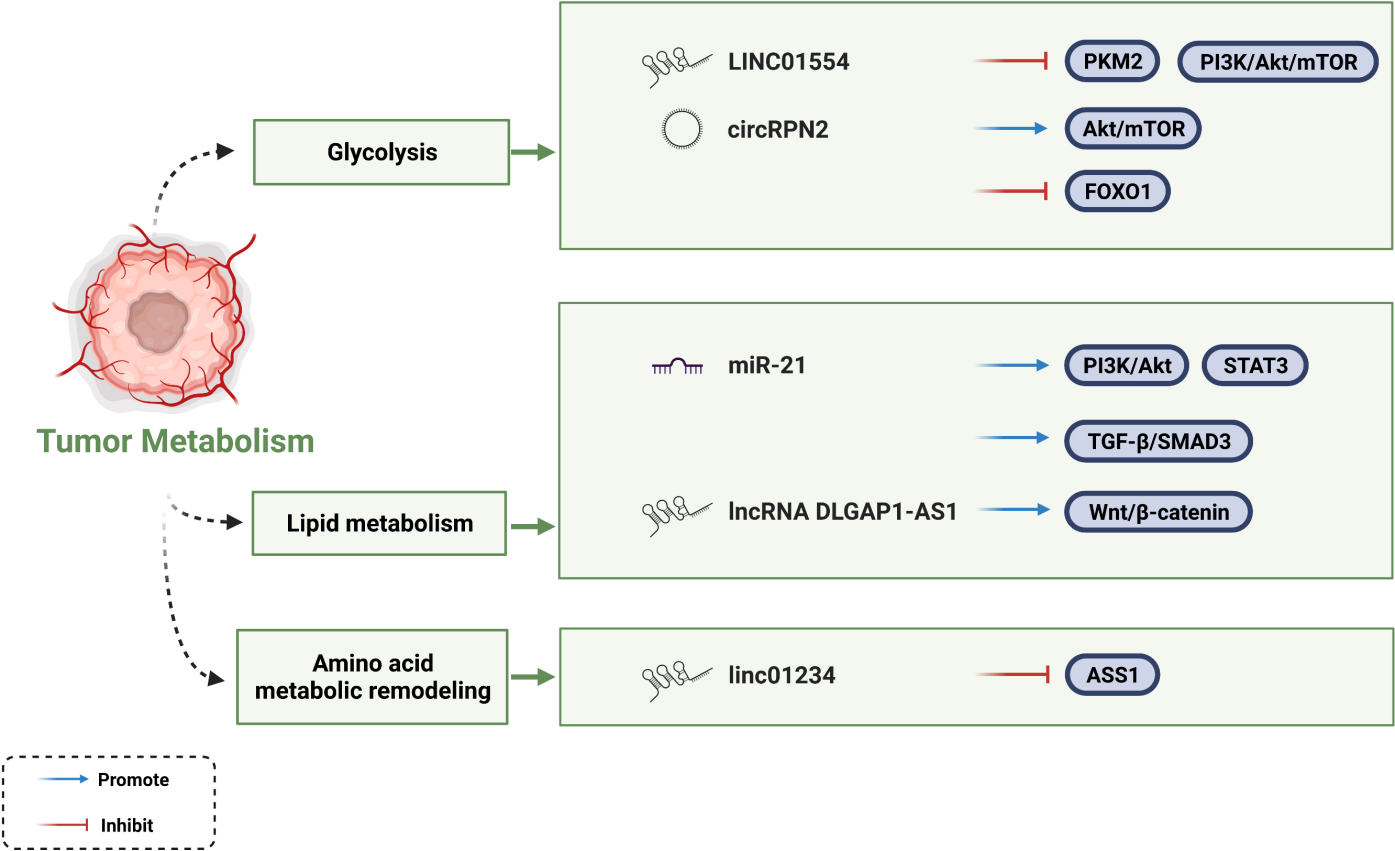
Regulatory mechanisms of ncRNAs to tumor metabolism.

Amino acid metabolic remodeling is another powerful proponent of cancer cell malignant activities. Mechanism can be attributed to the interaction between lncRNA and rate-limiting enzyme of amino acid synthesis pathway. Chen *et al.* found that LINC01234 bound to the promoter of ASS1 to inhibit its transcriptional activation, thereafter leading to increased aspartate levels and activation of rapamycin pathway targets (128). As the understanding of tumor cell metabolism in TME continues to advance, people have discovered multiple strategies to target tumor metabolism. Currently, fatty acid synthesis inhibitors (FAS), especially targeting fatty acid synthase (FASN), have been focused on as potential strategies for cancer treatment. The molecular mechanisms by which the ncRNAs mentioned in this section affecting HCC metabolism are summarized in **Figure 4**.

### Regulation of drug resistance

For unresectable HCC, the main treatment approaches include chemotherapy (e.g., Cisplatin and 5-Fluorouracil) and molecular targeted therapies (e.g., multi-kinase inhibitors, monoclonal antibodies, and ICIs). Multidrug resistance (MDR) is inevitable in tumor cells with their rapid development. Statistics indicate that only 30% of HCC patients show an increase in overall survival (OS) by 3 months after treatment with Sorafenib (129). Moreover, Sorafenib resistance was observed within 6 months of treatment, with adverse events of varying degrees, such as gastrointestinal reactions, hand-foot syndrome, and hypertension (130, 131). To further understand the emerging function and mechanism axis of ncRNAs in HCC, chemoresistance has become a hot research topic.

Aberrantly expressed miRNAs are universal features of HCC. From the perspective of clinical medication (Sorafenib, 5-Fluorouracil, Cisplatin), researchers have systematically elaborated on miRNAs modulating HCC drug resistance as well as the underlying mechanisms (132). Glucose metabolism has been implicated in the maintenance of Sorafenib resistance in HCC cells (133). Activation of the PI3K/Akt signaling pathway enhances glycolysis, and the expression of downstream glycolysis genes cause reactions in Sorafenib-resistant HCC cells. Studies verified that both miR-30a-5p and miR-32-5p were abnormally expressed in drug-resistant tissues, and may be targets for reversing Sorafenib resistance in HCC. miR-30a-5p induces MDR to activate the PI3K/Akt signaling pathway by upregulating CLCF1, while miR-32-5p activates the PI3K/Akt signaling pathway by downregulating PTEN (133, 134). Intravenous injection of miR-199-modified vehicle suppress mTOR signaling (135, 136). Overactivation of the mTOR signaling pathway promotes tumorigenesis and tumor progression, which significantly sensitizes HCC cells to Doxycycline (137).

Autophagy plays a dual role in drug resistance. Few lncRNAs influence Sorafenib-induced chemoresistance and sensitivity through autophagy mechanism. A previous study showed that Rutin treatment reduced the number of autophagosomes in Sorafenib-resistant cells, while reducing the expression of the lncRNA BANCR (BANCR knockdown increases the sensitivity of Sorafenib-resistant cells to Sorafenib). The lncRNA BANCR inhibits autophagy through the BANCR/miRNA-590-5P/OLR1 axis (138). Another study reported that the lncRNA CRNDE has the opposite effect as it enhances the stability of ATG4B primarily by isolating miR-543, thereby triggering autophagy (139). The autophagy mechanism of drug resistance is regulated not only by lncRNAs but also by circRNAs. Li *et al.* identified 968 dysregulated circRNAs in Cisplatin-resistant HCC tissues and reported that circARNT2 competes with miR-155-5p to upregulate PDK1-induced autophagy, ultimately enhancing the sensitivity of HCC cells to Cisplatin (140).

**Figure 5:**
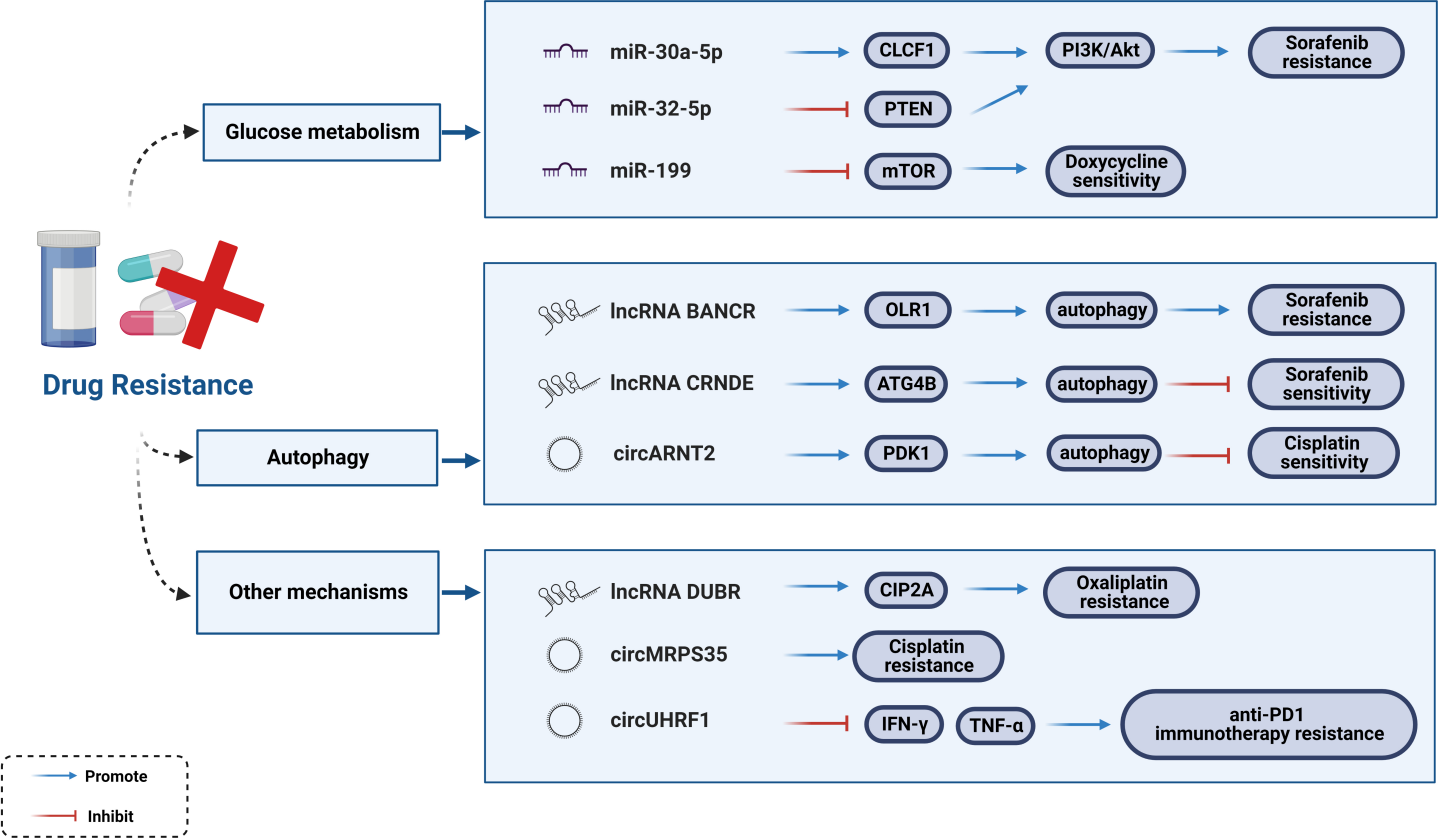
Regulatory mechanisms of ncRNAs to drug resistance.

In addition to autophagy, circRNAs function through many other mechanisms. For instance, circMED27, circMEMO1, circFOXM1, circFN1, and circRNA-SORE act as ceRNAs targeting corresponding miRNAs to affect the drug resistance or sensitivity of HCC cells to chemotherapy drugs (140–144). Existing studies have confirmed the molecular mechanisms associated with resistance to the first-line chemotherapy drug, Oxaliplatin (OXA), in which cancer stem cells (CSCs) play an important role (145, 146). The lncRNA DUBR is highly expressed in liver CSCs and functions as one of the factors that promote OXA resistance. Liu *et al.* found that the specificity protein 1 (SP1)-induced lncRNA DUBR upregulates CIP2A expression *via* the E2F1 protein, promoting stemness and OXA resistance. They also identified another mechanism by which lncRNA DUBR acts as a ceRNA to upregulate CIP2A, which in turn stabilizes the E2F1 protein, thereby activating the Notch1 signaling pathway (147). Additionally, circMRPS35-encoding peptide is significantly induced by chemotherapeutic agents and promotes Cisplatin resistance, suggesting that circMRPS35 may be a possible mediator of Cisplatin resistance (148). Interestingly, emerging evidence suggests that ncRNAs are involved in the resistance to immune checkpoint blockers. A newly reported example is that where circUHRF1 can impair the function of NK cells and induce an exhausted phenotype that cannot secrete IFN-γ and TNF-α. Hence, circUHRF1 could be regarded as a therapeutic target for anti-PD1 immunotherapy and drug resistance (69). In conclusion, clarifying the mechanisms of drug resistance (**Figure 5**) and targeting these dysregulated ncRNAs would be a fatal step forward in HCC treatment.

## Clinical significance of ncRNAs in HCC

**Figure 6:**
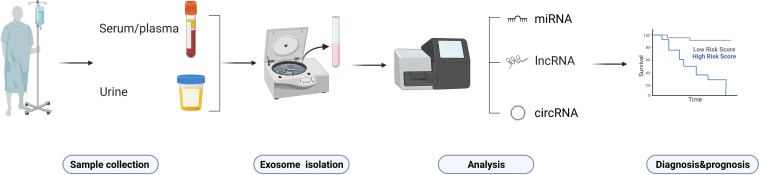
Procedure of ncRNAs as non-invasive biomarkers.

Most HCC patients are often diagnosed too late or have a high recurrence rate, that is why exploring predictive/prognostic biomarkers in early-stage of HCC is relevant for physicians to develop precise treatment strategies (149). Evaluation of tissues from liver biopsies and surgical specimens are both invasive approaches that too painful for the patient. α-fetoprotein (AFP) is the “gold standard” but with suboptimal sensitivity and specificity of only 39-64% and 76-91% (150, 151). Moreover, AFP appears as an unanticipated false positive that is elevated in some patients with chronic liver diseases (e.g., cirrhosis, viral hepatitis, etc.). More reliable biomarkers are urgently needed to monitor and diagnose HCC and improve patient prognosis. The secretion of circulating ncRNAs has been identified in biological fluids (e.g., serum, plasma, and urine) of patients (the process is shown in **Figure 6**), where changes in levels or expression indicate cancer status. Owing to their abundance, accessibility, non-invasiveness, easy reproducibility, and disease specificity, ncRNAs are considered ideal non-invasive diagnostic biomarkers for HCC (152). As a non-invasive liquid biopsy, ncRNAs have entered the spotlight for the development of diagnostic markers in oncology. Several ncRNAs in body fluids have been the most promising biomarkers to date. This section summarizes the main diagnostic/prognostic markers currently under investigation.

### ncRNAs act as diagnostic biomarkers

#### miRNAs

In recent years, several research groups have evaluated the potential of miRNAs as diagnostic, prognostic, and therapeutic responsiveness biomarkers for liver disease using clinical trial databases or clinical trials. In addition, the clinical utility of circulating miRNAs in patients with HCC has been explored, such as miR-21, miR-122, miR-96, miR-194/192, and miR-484; more information is summarized in **Table 1**. The plasma oncogenic factor miR-21 has been explored as a biochemical marker for HCC considering its important role in HCC progression. The potential of miR-21 as a biomarker for HCV patients complicated by HCC was explored by collecting trials registered in the *clinicaltrial.gov* database, enrolling a total of 100 participants aged 31-67 years in Egypt (*NCT05449847*). miR-21 can differentiate between uncomplicated and complicated HCV patients. It has been suggested that multiple miRNAs or a combination of miRNAs with the already widely used AFP may be more desirable diagnostic modalities. For instance, the combination of miR-21, miR-122, and miR-96 in serum showed a much higher diagnostic accuracy in the cirrhotic group than AFP or miR-21 alone (153). A combination of miRNAs has shown a higher area under the curve (AUC) value of 0.924. Thus, miR-21 was not only superior to AFP alone, but also showed better differentiation in combination with AFP. Studies have confirmed that miR-221 is also upregulated in patients with HCC. The AUC of the combination with AFP for diagnosis is 0.945, sensitivity is 93.33%, specificity is 77.78%, accuracy is 90.9%, and thus the combination has a better performance than individual detection (164). Clinical trials of miR-221 have also been conducted, and details can be found by querying *NCT02928627*. Shohda identified liver-specific miR-484 as an early diagnostic marker for HCC. miR-484 has shown great sensitivity in distinguishing HCC from non-HCC, with an AUC of 0.67. Moreover, miR-484 signatured across various stages of HCV-mediated hepatic disease progression, revealing promising performance in staging, prognosis, and early diagnosis (157).

**Table 1:** Diagnostic biomarkers of ncRNAs for HCC.

Types	ncRNA	Sample	Expression	Patients enrolled	Diagnostic accuracy(PC vs. non-PC)	Confidence Interval (CI)	References
miRNA	miR-93-5p	urine	↑	64 HCC, 65 HC	AUC 0.906, sensitivity 87.9%, specificity 93.8%	/	(153, 154)
miRNA	miR-93-5p	plasma	↑	64 HCC, 65 HC	AUC 0.905, sensitivity 86.2%, specificity 95.4%	/	(154)
miRNA	miR-155	serum	↑	80 HCC, 80 CHB, 40 HC	AUC 0.743, sensitivity 80%, specificity 62.5%	/	(155)
miRNA	miR-21+miR-122+miR-96	plasma	↑	50 HCC, 50 LC, 50 HC	AUC 0.924, sensitivity 82%, specificity 92%	/	(153)
miRNA	miR-10b-5p	plasma	↑	38 HCC	AUC 0.65, sensitivity 76%, specificity 55%	0.54-0.77	(156)
miRNA	miR-221-3p	plasma	↑	38 HCC	AUC 0.69, sensitivity 87%, specificity 52%	0.58-0.80	(156)
miRNA	miR-21-5p	plasma	↑	38 HCC	AUC 0.78, sensitivity 74%, specificity 77%	0.69-0.87	(156)
miRNA	miR-223-3p	plasma	↑	38 HCC	AUC 0.63, sensitivity 61%, specificity 70%	0.51-0.75	(156)
miRNA	miR-484	plasma	↓	41 HCC, 47 HF, 40 LC, 40 HC	AUC 0.67	0.5067–0.8307	(157)
miRNA	miR-224	serum	↑	89 HCC, 50 HC	AUC 0.910	0.84-0.98	(158, 159)
miRNA	miR-148a	plasma	↓	155 HCC, 95 LC, 95 HC	AUC 0.949, sensitivity 90.6%, specificity 92.6%	0.916-0.981	(160, 161)
miRNA	miR-409-3p	serum	↓	20 HCC,	AUC 0.80, sensitivity 85%, specificity 70%	0.66-0.95	(162)
miRNA	miR-125a-3p	serum	↓	12 HCC	AUC 0.98, sensitivity 80%, specificity 100%	/	(158, 163)
miRNA	miR-221	serum	↑	45 HCC, 45 HC	AUC 0.945, sensitivity 93.33%, specificity 77.78%	0.655-0.894	(154, 164)
miRNA	miR-221+AFP	serum	↑	45 HCC, 45 HC	sensitivity 96.49%, specificity 88.00%	/	(164)
miRNA	miR-125b	plasma	↓	64 HCC, 59 LC, 63 CHB, 56 HC	AUC 0.891, sensitivity 85.9%, specificity 78.6%	0.835-0.947	(156, 165)
miRNA	miR-122	plasma	↓	80 HCC, 20 HC	AUC 0.98, sensitivity 87.5%, specificity 95%	/	(161)
miRNA	miR-224	plasma	↑	80 HCC, 20 HC	AUC 0.93, sensitivity 92.5%, specificity 90%	/	(161)
miRNA	miR-338-5p	plasma	↑	47 HCC, 29 LC, 31 HC	AUC 0.909, sensitivity 72.3%, specificity 99.68%	/	(166)
miRNA	miR-764	plasma	↑	47 HCC, 29 LC, 31 HC	AUC 0.791, sensitivity 74.5%, specificity 77%	/	(166)
miRNA	miR-15b-5p	plasma	↑	47 HCC, 29 LC, 31 HC	AUC 0.765, sensitivity 68.1%, specificity 80%	/	(166)
miRNA	miR-21	plasma	↑	126 HCC, 50 HC	AUC 0.953, sensitivity 87.3%, specificity 92.0%	/	(159, 164)
lncRNA	SCARNA10	serum	↑	182 HCC, 105 BLD, 149 HC	AUC 0.82, sensitivity 70%, specificity 77%	/	(167)
lncRNA	SCARNA10+AFP	serum	↑	182 HCC, 105 BLD, 149 HC	AUC 0.92, sensitivity 88%, specificity 80%	/	(167)
lncRNA	HULC,	serum	↑	129 HCC, 49 LC, 27 CHB, 93 HC	AUC 0.796, sensitivity 86.0%, specificity 62.4%	0.734-0.858	(168)
lncRNA	MALAT1	serum	↑	129 HCC, 49 LC, 27 CHB, 93 HC	AUC 0.768, sensitivity 59.7%, specificity 80.6%	0.706-0.830	(168)
lncRNA	LINC00152	serum	↑	129 HCC, 49 LC, 27 CHB, 93 HC	AUC 0.895, sensitivity 78.3%, specificity 89.2%	0.854-0.936	(168)
lncRNA	PTENP1	serum	↓	129 HCC, 49 LC, 27 CHB, 93 HC	AUC 0.602, sensitivity 89.1%, specificity 29.0%	0.526-0.678	(168)
lncRNA	PTTG3P	serum	↑	129 HCC, 49 LC, 27 CHB, 93 HC	AUC 0.785, sensitivity 82.9%, specificity 61.3%	0.723-0.847	(168)
lncRNA	SPRY4-IT1	serum	↑	129 HCC, 49 LC, 27 CHB, 93 HC	AUC 0.808, sensitivity 76.7%, specificity 71.0%	0.750-0.866	(168)
lncRNA	UBE2CP3	serum	↑	129 HCC, 49 LC, 27 CHB, 93 HC	AUC 0.812, sensitivity 88.4%, specificity 62.4%	0.754-0.870	(168)
lncRNA	UCA1	serum	↑	129 HCC, 49 LC, 27 CHB, 93 HC	AUC 0.858, sensitivity 81.4%, specificity 75.3%	0.810-0.907	(168)
lncRNA	Linc00152+AFP	serum	↑	129 HCC, 49 LC, 27 CHB, 93 HC	AUC 0.906 sensitivity 85.3%, specificity 83.4%	0.870-0.942	(168)
lncRNA	Linc00152+UCA1+AFP	serum	↑	129 HCC, 49 LC, 27 CHB, 93 HC	AUC 0.912, sensitivity 82.9%, specificity 88.2%	0.878-0.945	(168)
lncRNA	SENP3-EIF41	plasma	↓	3 HCC, 3 HC	AUC 0.8028	/	(169)
lncRNA	LINC00853	serum	↑	90 HCC, 35 LC, 28 CHB, 29 HC	AUC 0.934, sensitivity 93.75%, specificity 89.77%	0.887-0.966	(170)
lncRNA	lnc85	serum	↑	112 HCC, 43 LC, 52 HC	AUC 0.869, sensitivity 80.0%, specificity 76.5%	0.828-0.918	(171)
lncRNA	lncRNA-D16366	serum	↓	107 HCC, 28 HBV, 18 ALD, 12 fatty liver disease, 85 HC	AUC 0.752, sensitivity 65.5%, specificity 84.6%	/	(172)
lncRNA	lncRNA SNHG1	plasma	↑	72 HCC, 50 LC, 50 HC	AUC 0.92,	0.86-0.96	(173)
lncRNA	GAS5-AS1	plasma	↓	156 HCC, 58 HC	AUC 0.824, sensitivity 89.5%, specificity 89.5%	0.741-0.906	(174)
lncRNA	lncRNA LRB1	serum	↑	326 HCC, 73 HC	AUC 0.892, sensitivity 92.43%, specificity 71.85%	0.843-0.922	(175)
lncRNA	LncRNA UCA1+lncRNA WRAP53+AFP	serum	↑	82 HCC, 34 CHC, 44 HC	100% sensitivity	/	(176)
lncRNA	lncRNA PVT1+uc002mbe.2+ and AFP	serum	↑	71 HCC, 64 HC	AUC 0.764, sensitivity 60.56%, specificity 90.62%	0.684–0.833	(177)
circRNA	hsa_circ_0005397	plasma	↑	89 HCC, 40 BLD, HC 79	AUC 0.737, sensitivity 75.36%, specificity 66.67%	0.671-0.795	(178)
circRNA	circRNA 0006602	plasma	↑	87 HCC, 30 HC	AUC 0.907, sensitivity 77.0%, specificity 93.3%	/	(179)
circRNA	Circ-CDYL+HDGF+HIF1AN	/	↑	/	AUC 0.73, sensitivity 75.36%, specificity 66.67%	0.65-0.80	(180)
circRNA	circTMEM45A	serum	↑	30 HCC, 30 HC	AUC 0.888	0.823-0.954	(181)
circRNA	hsa_circ_0051443	plasma	↓	3 HCC, 3 HC	AUC 0.8089	/	(182)
circRNA	hsa_circ_0000976	plasma	↑	158 HCC, 52 CHB, 50 LC, 53 HC	AUC 0.863	0.819–0.907	(183)
circRNA	hsa_circ_0007750	plasma	↑	152 HCC, 54 CHB, 50 LC, 50 HC	AUC 0.843	0.796–0.890	(183)
circRNA	hsa_circ_0139897	plasma	↑	290 HCC, 80 CHB, 80 LC, 76 HC	AUC 0.769	0.728–0.810	(183)
circRNA	circRNA SMARCA5	plasma	↓	133 HCC, 31 LC, 33 HC	AUC 0.938, sensitivity 86.67%, specificity 89.32%	0.910-0.966	(184)
circRNA	circRNA SMARCA5+AFP	plasma	↓	133 HCC, 31 LC, 33 HC	AUC 0.992, sensitivity 100%, specificity 100%	0.983-1.002	(184)
circRNA	hsa_circ_0003998	plasma	↑	100 HCC, 50 CHB, 50 HC	AUC 0.894, sensitivity 84.0%, specificity 80%	0.86-0.922	(185)
circRNA	hsa_circ_0001445	plasma	↓	104 HCC, 57 LC, 44 CHB, 52 HC	AUC 0.862, sensitivity 94.2%, specificity 71.2%	0.710-0.845	(186)
circRNA	circ_104075	serum	↓	10 HCC, 60 HC	AUC 0.973, sensitivity 96%, specificity 98.3%	/	(187)

CHB, chronic hepatitis B; LC, liver cirrhosis; HC, healthy control. ↑ represents the expression are upregulated in samples; ↓ represents the expression are upregulated in samples; / represents cannot find data in references.

#### LncRNAs

LncRNA UCA1 and lncRNA WRAP53 act as natural p53 single transcripts and are effective in regulating the expression of corresponding sense genes. The role of lncRNA UCA1 in bladder cancer and breast cancer has been previously identified, while the role for HCC remains elusive. In a study, both lncRNAs were found to be highly expressed in the sera of HCV patients with HCC, with AUC-ROC of 0.76 and 0.87, respectively. Interestingly, the combination of lncRNA UCA1, lncRNA WRAP53, and AFP resulted in a high diagnostic sensitivity of 100%, strongly confirming the diagnostic efficacy of the combination of miRNA and AFP (176). To validate these two lncRNAs as potential biomarkers for HCC diagnosis, a clinical trial has been published, enrolling a total of 80 participants. This work is nearing completion and we will wait to see the results of the validation (*NCT05088811*). In another study, Huang discussed the diagnostic efficacy of eight lncRNAs alone and in combination with AFP, in which the combination of LINC00152 and AFP in serum had the highest accuracy in predicting HCC. The AUC and sensitivity of LINC00152 alone are limited and correspond to an increase when used in combination with AFP. Next, researchers have investigated the diagnostic ability of the combinations of various lncRNAs and AFP, and the combination of LINC00152, UCA1, and AFP has shown the most reliable predictive ability, with an AUC of 0.912, sensitivity of 82.9%, and specificity of 88.2% (168). The expression of SENP3-EIF4A1 in patients with HCC was significantly lower than that in healthy controls, with an AUC of 0.8028 obtained by ROC analysis. Simultaneously, its expression is associated with tumor size, tumor stage, and lymph node metastasis (169). The ROC curve showed that LINC00853 exhibited excellent discriminatory ability in the diagnosis of all-stage HCC (AUC = 0.934, 95% CI = 0.887-0.966). On comparing the diagnostic performance with that of AFP with a 14-fold cut off value, LINC00853 showed sensitivity, specificity, and positive predictive values of 93.75%, 89.77%, and 76.92% respectively. These parameters all far exceed the sensitivity of 9.38%, specificity of 72.73%, and positive predictive value of 11.11% exhibited by AFP for diagnosis of early-stage HCC (170). The combination of lncRNA PVT1, uc002mbe.2, and AFP also far exceeded either the lncRNA or AFP assessed alone. Surprisingly, this combination distinguished patients with HCC from healthy controls, regardless of whether the patients were infected with HBV (177).

#### CircRNAs

Compared to miRNA and lncRNA, the number of studies on circRNA as a diagnostic biomarker is relatively few. Plasma circSMARCA5 can differentiate liver disease progression with high accuracy (AUC = 0.938, sensitivity of 0.853, specificity of 0.711). More excitingly, it has significant implications in diagnosing HCC patients with low AFP levels, especially for those with serum levels below 200 ng/ml, and is a better serum predictor for patients with poorly diagnosed AFP (184). Circ_104075 is highly expressed in patients with HCC and levels decreased after curative surgery. It is positively correlated with stage of HCC and was able to predict the development of disease. It has an AUC of 0.973, sensitivity of 96.0%, and specificity of 98.3%, surpassing the classical protein biomarkers AFP, α-fetoprotein-L3 (AFP-L3), and des-carboxy-prothrombin (DCP) (187). Likewise, circRNA applies to this combination. Circ-CDYL, the most significantly upregulated circRNA in a ncRNA network from a validated tumor tissue, interacts with the target genes encoding hepatoma-derived growth factor (HDGF) and hypoxia-inducible factor asparagine hydroxylase (HIF1AN). These proteins are highly and specifically expressed in early-stage of HCC. Compared with early-stage HCC patients, the combination of Circ-CDYL, HDGF, and HIF1AN increased the AUC, sensitivity, and specificity to 0.73, 75.36%, and 66.67% respectively. These results confirmed that the diagnostic efficiency of circ-CDYL or circ-CDYL in combination with HDGF and HIF1AN was higher than that of AFP alone, however this does not apply to advanced HCC (180). Along with the proliferation of research on circRNAs, circRNAs that can be used as diagnostic biomarkers also containing circTMEM45A, hsa_circ_0051443, hsa_circ_0000976, hsa_circ_0007750, and hsa_circ_0139897 (181–183).

### ncRNAs act as prognostic biomarkers

HCC is known for its high recurrence rate and poor prognosis. In addition to their diagnostic values, circulating ncRNAs have been identified as valid prognostic markers. When changes occur in the human body, the altered levels of these ncRNAs can be used as a feature and a “beacon” for cancer prognosis. Studies on the application of ncRNAs as prognostic biomarkers in hepatocellular carcinoma are summarized in **Table 2**.

**Table 2:** Prognostic biomarkers of ncRNAs for HCC.

Types	ncRNA	Sample	Expression	Patients enrolled	Associated factors and clinicopathological characteristics	References
miRNA	miR-484	serum	↑	/	OS, PFS	(149, 188, 189)
miRNA	miR-34a	serum	↓	60 HCC, 60 HC	OS, differentiation degrees, TNM stage, tumor invasion depth, lymph node metastasis, and vascular invasion	(189, 190)
miRNA	miR-21	serum	↑	/	OS, PFS, TNM stage, T stage and portal vein thrombosis	(149, 188)
miRNA	miR-122	whole blood	↑	54 HCC, 28 LC, 12 HC	PFS	(191)
miRNA	miR-497	serum	↓	50 HCC, 50 HC	differentiation degrees, TNM stage, and metastasis	(192)
miRNA	miR-1246	serum	↑	50 HCC, 50 HC	differentiation degrees, TNM stage, and metastasis	(192)
miRNA	miR-92a-3p	plasma	↑	42 HCC	OS, DFS	(107)
miRNA	miR-4454	serum	↓	86 HCC (40 curative treatment, 46 TACE)	OS, DFS	(193)
miRNA	miR-4530	serum	↓	86 HCC (40 curative treatment, 46 TACE)	OS, DFS	(193)
miRNA	miR-122	plasma	↑	112 HCC	tumor number, tumor size, TFS	(194)
miRNA	miR-122	plasma	↑	120 HCC	OS, DFS, TNM stage	(195)
miRNA	miR-122	serum	↑	122 HCC	OS	(191, 196)
miRNA	miR-139	plasma	↓	31 HCC, 31 HC	OS	(193, 197)
lncRNA	lncRNA SCARNA10	serum	↑	182 HCC, 105 BLD, 149 HC	tumor size, differentiation degrees, TNM stage, vascular invasion, and metastasis	(167, 190)
lncRNA	lncRNA CRNDE	serum	↑	166 HCC, 100 HC	tumor size, tumor differentiation, and TNM stage	(192, 198)
lncRNA	LINC00853	serum	↑	90 HCC, 35 LC, 28 CHB, 29 HC	OS in mUICC stage II	(170, 192)
lncRNA	lncRNA-ATB	serum	↑	79 HCC	OS, PFS, TNM stage, tumor size, CRP, T stage, portal vein thrombosis	(107, 149)
lncRNA	lncRNA-D16366	serum	↓	107 HCC, 28 HBV, 18 ALD, 12 fatty liver disease, 85 HC	tumor size, HbsAg, portal vein tumor thrombus, Child-Pugh score	(172, 197)
lncRNA	lncRNA X91348	serum	↓	107 HCC, 82 HC	OS, tumor size, HBsAg, and Child-Pugh	(199)
lncRNA	lncRNA LRB1	serum	↑	326 HCC, 73 HC	OS, AFP expression, tumor size, tumor stage, and venous invasion	(175)
lncRNA	LINC00635	serum	↑	60 HCC, 85 LC, 96 CHB, HC 60	OS, lymph node metastasis, and TNM stage	(200, 201)
lncRNA	lncRNA RP11-466I1.1	serum	↑	83 HCC	tumor size, cirrhosis, and histological grade	(199, 202)
lncRNA	lncRNA UCA1	serum	↑	70 HCC	RFS, median follow up period	(170, 203)
lncRNA	lncRNA c-JUN	serum	↑	70 HCC	RFS, median follow up period	(203, 204)
lncRNA	lncRNA MVIH	serum	↑	215 HCC	OS, RFS	(149, 200)
circRNA	circ_0000437	serum	↑	100 HCC, 100 HC	TNM stage, differentiation degree, tumor size, and BCLC stage	(175, 205)
circRNA	hsa_cic_0005397	plasma	↑	89 HCC, 40 benign liver diseases, 79 HC	OS, tumor size, and TNM stage	(178, 198)
circRNA	circETFA	plasma	↑	56 HCC	OS, cell cycle arrest	(201, 206)
circRNA	circUHRF1	plasma	↑	/	tumor size, microvascular invasion	(69, 167)
circRNA	circ-FOXP1	serum	↑	30 HCC, 16 HC	TNM stage, and microvascular invasion	(202, 207)
circRNA	hsa_circ_0003998	plasma	↑	100 HCC, 50 CHB, 50 HC	OS, tumor size, vascular invasion, differentiation degree,	(185, 203)
circRNA	hsa_circ_0064428	plasma	↑	120 HCC	OS, tumor size	(203, 208)
circRNA	circ-ZEB1.33	serum	↑	64 HCC, 30 HC	OS, tumor size, TNM stage	(69, 209)

CHB, chronic hepatitis B; LC, liver cirrhosis; HC, healthy control; HBV, hepatitis B virus; ALD, alcoholic liver disease; BLD, benign liver disease; TACE, transcatheter arterial chemoembolization; OS, overall survival; PFS, progression-free survival; TNM stage, tumor, node, and metastasis stage; DFS, disease-free survival; TFS, transplantation-free survival; mUICC, modified Union for International Cancer Control; CRP, C-reactive protein; HBsAg, Hepatitis B surface antigen; RFS, relapse-free survival; BCLC stage, Barcelona Clinic Liver Cancer stage. ↑ represents the expression are upregulated in samples; ↓ represents the expression are upregulated in samples; / represents cannot find data in references.

#### miRNAs

Differentially expressed serum/plasma miRNAs are helpful in the prognosis of HCC. Circulating miR-21 has long been shown to be an oncogenic factor, with its high serum levels predicting poor prognosis. Extensive studies have associated it with poor survival, high levels of recurrence, and an increased risk of disease progression. According to Lee’s study, miR-21 not only correlates with OS and progression-free survival (PFS), but also relates to multiple prognostic factors such as tumor, nodes, and metastases (TNM) stage, T stage, and portal vein thrombosis (149) (188). When using the TNM stage system, miR-21 expression was higher in stages III and IV than in stages I and II; when using the BCLC staging system, miR-21 expression was higher in late BCLC stages C-D than in early BCLC stages 0-B (149). Another study showed that elevated miR-484 in serum could predict HCV-induced liver lesion progression and is strongly associated with shorter OS and PFS (189). In addition, increased levels of miR-122 in serum, predict a longer survival rate (196). By testing blood samples from 50 each of HCC patients, cirrhotic patients and healthy volunteers, miR-122 was found to be less expressed in HCC patients, while miR-21 and miR-96 were opposite. The expression of all three affected the survival time of patients. Interestingly, the combination of the three was better than each miRNA alone in predicting the survival time of patients (153). Unlike the majority of reports published, miR-21 were measured in whole blood samples rather than in serum or plasma in Pelizzaro’s study (191). In a study, a KM survival analysis showed that high expression of miR-4454 and miR-4530 was significantly associated with improved OS (193). The above evidence confirms that miRNAs are potential prognostic markers of HCC.

#### LncRNAs

LncRNA MVIH, a microvascular invasion-associated lncRNA, is highly expressed in HCC. Sheng ***et al.*** first identified the relationship between up-regulated lncRNA MVIH expression and specific clinicopathological characteristics. Kaplan-Meier’s analyses of correlations between lncRNA MVIH expression and RFS and OS of 215 HCC patients after hepatectomy indicated that lncRNA MVIH is an independent risk factor for RFS and OS (200). The expression of lncRNA X91348 in patients with HCC was significantly lower than that in healthy individuals. A total of 107 HCC patients and 82 age- and sex-matched healthy volunteers were included in this study. Clinicopathological characteristics such as tumor size, HBsAg, and Child-Pugh could be observed influence the expression of X91348. The relationship between X91348 expression and survival was assessed after 5 years of follow-up. A median follow-up rate of 31.02 ± 15.11 months for patients with HCC was obtained, and the OS of patients with high X91348 expression was longer than that for patients with low expression. In conclusion, X91348 has a satisfactory prognostic ability (199). LINC00853 serves not only as a diagnostic biomarker but also as a prognostic marker in this cohort. High LINC00853 expression predicted lower OS in modified Union for International Cancer Control (mUICC) stage II and was independent of other stages of HCC (170). High expression of HOTTIP in the blood is an independent prognostic factor for tumor recurrence after liver transplantation, suggesting a short OS (204). Higher serum levels of circulating lncRNA-ATB could be associated with OS, PFS, TNM stage, tumor size, C-reactive protein (CRP), T stage, and portal vein thrombosis (149, 195). Moreover, a variety of lncRNAs have been investigated in **Table 2**.

#### CircRNAs

Stably expressed in the plasma, hsa_cic_0005397 has an intact closed-loop structure. According to a survival analysis based on follow-up data, hsa_cic_0005397 may serve as an independent prognostic marker for OS, as evidenced by the positive correlation between tumor size and TNM stage (178). Regarding circRNAs, the high expression of circUHRF1 in the plasma is associated with large tumor size and high microvascular invasion, which indicates the potential of circUHRF1 in predicting poor prognosis (69). Clinically, high expression of circ-FOXP1 and Circ-ZEB1.33 in serum is strongly associated with large tumors, advanced TNM, and poor prognosis (207, 209). *In vitro* experiments have also confirmed that circ-ZEB1.33 and circETFA affect tumor cell proliferation by regulating the cell cycle, likely serving as a novel prognostic marker (206). Circ_0000437 expression was positively correlated with TNM classification, differentiation degree, tumor size, and Barcelona Clinic Liver Cancer (BCLC) stage; hsa_circ_0003998 expression positively correlated with serum AFP level, tumor diameter, and microvascular invasion, whereas hsa_circ_0064428 expression was negatively correlated with patient’s survival, tumor size and metastasis. To date, fewer circRNAs derived from plasma or serum have been used as prognostic markers compared with those obtained from tissues. In summary, the prognostic information for circRNAs derived from body fluids remains to be explored.

In summary, ncRNAs can be valuable biomarkers for the diagnosis and prognosis of HCC. However, the current problem is that studies about ncRNAs are scarce as diagnostic and prognostic biomarkers (especially those derived from blood and urine) (149). The heterogeneity of tumors in different populations is a significant challenge for their application. Therefore, future multicenter and large-scale diagnostic/prognostic nested case–control studies are required to validate their utility. Fortunately, institutions have begun to validate the potential of certain ncRNAs as markers clinically, such as miR-21, miR-221, lncRNA UCA1, and lncRNA WRAP53 in the clinical trials *NCT05449847, NCT02928627, NCT05088811* mentioned above. Although some trials are not yet completed, these are “milestones” for ncRNAs as clinical biomarkers for HCC. It is also believed that more hospitals, research institutions and companies will be involved in the exploration and development of clinical biomarkers.

### Methods for detecting ncRNAs in clinical samples

Detecting tumors in their early stage is a long-standing goal of researchers. In the past few years, the study of ncRNAs in body fluids as potential biomarkers has grown exponentially. However, no ncRNAs have yet been able to be used clinically to detect hepatocellular carcinogenesis and predict prognosis. The limitations of circulating ncRNA measurement techniques are the main reason for this dilemma. There are several main assays available, corresponding to different advantages and limitations, to detect miRNA as an example: (1) qRT-RCR has the advantage of being widely available and highly sensitive, but has the disadvantage of having several biological and technical limitations. First, it is limited to quantifying only a defined set of miRNAs and cannot be used for high-throughput analysis. Second, because of the low abundance in biofluids, genomic DNA contamination needs to be removed prior to the reverse transcription step (210, 211). (2) Assessing the relative number of miRNAs by comparing the fluorescence intensity emitted by microarrays with hundreds of thousands of probes with labeled miRNAs has the advantage of high throughput, but the resulting disadvantage of a fixed range and the inability to detect new unannotated miRNAs. Therefore, this is only suitable for preliminary screening (212, 213). (3) Next Generation Sequencing (NGS) based on deep sequencing to detect circulating miRNAs not only overcomes the shortcomings of microarrays that can only detect known miRNAs, but also greatly increases in the detection order of magnitude. The cost of sequencing is reduced, while more information can be harvested. However, bioinformatics expertise is required for interpretation, and the technology is expensive with long turnaround time (214, 215). For ncRNAs, firstly their short sequences, showing higher levels of homology pose a significant challenge for accurate identification. Secondly, the low abundance predicts that the detection needs to span four orders of magnitude dynamic range with high sensitivity and accuracy requirements. Then, different detection methods correspond to different strengths and weaknesses, requiring researchers to break through and validate with newer studies. Notably, further efforts are required in this field.

## Therapeutic potential of ncRNAs in HCC

Protein-like targets with stable structures and conformations have occupied an absolute leadership position in human diseases as mainstream targets for drug development (216). However, less than 2% of RNAs are translated into proteins, and most proteins are “non-druggable” (217, 218). With the elucidation of the mechanisms by which ncRNAs affect diseases, the feasibility of targeting RNA continues to be demonstrated. ncRNA can regulate signaling pathways and affect different enzymes and genes. Having such a large and fine regulatory mechanism, ncRNAs are therefore at the core of a multi-target regulatory network. Data show that representative small-molecule drugs, such as Bisphenol-A, Mitoxantrone, and Enoxacin, act against different cancers by targeting ncRNAs, thus providing new insights for drug development (216).

**Figure 7:**
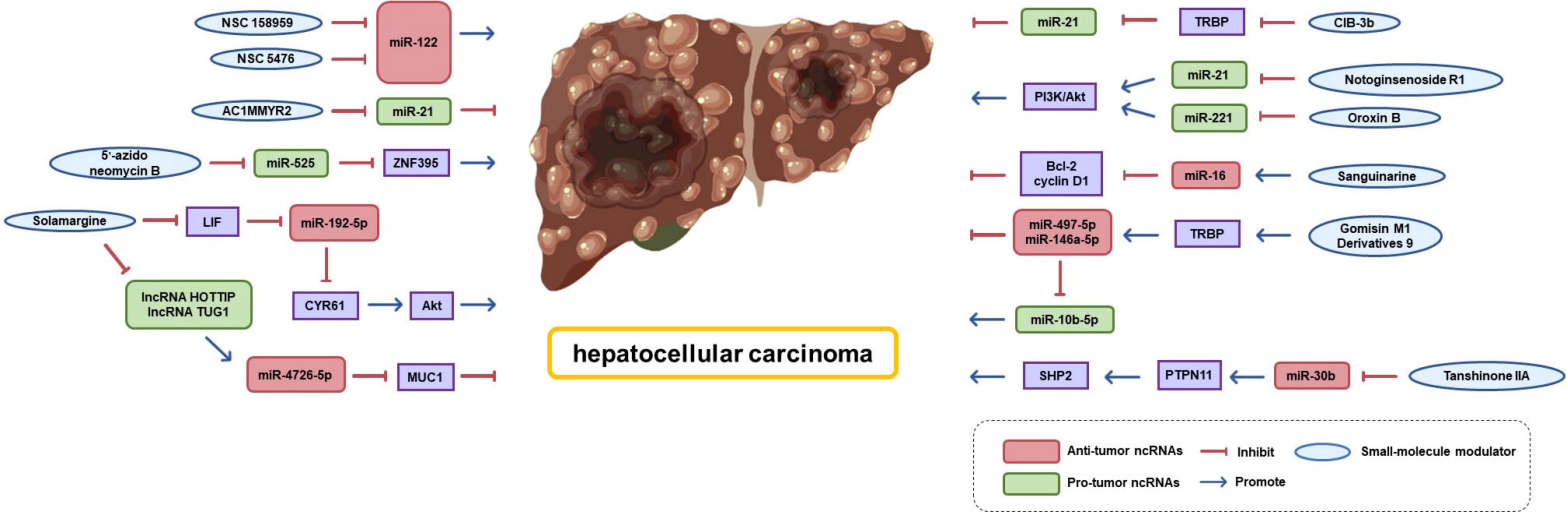
Small-molecule modulators exhibiting therapeutic activity against HCC that target miRNAs.

As shadow pioneers of ncRNAs, many small-molecule modulators that target miRNAs have been found to exhibit therapeutic activity against HCC (shown in **Figure 7**). Several small-molecule modulators that block miRNA biogenesis by directly binding to Drosha/Dicer-binding sites in pri/pre-miRNAs have been identified. For example, Douglas reported the first small-molecule modulator of miR-122 (the most abundant miRNA in the liver), demonstrating that small-molecule modulators of miRNA function have therapeutic potential. They found that two small-molecule inhibitors (NSC 158959 and NSC 5476) accelerated the processing and maturation of pri-miR-122 to miR-122 and were able to reduce hepatitis C virus replication (219). miR-525 confers invasive properties to HCC cells, while 5′-azido-neomycin B binds the Drosha processing site in pre-miR-525 to inhibit the production of mature miR-525 and salvage the expression of ZNF395 (220). Shi *et al.* screened AC1MMYR2, a small-molecule inhibitor of miR-21, using a high-throughput approach. They found that it blocks miR-21 maturation. AC1MMYR2 can reverse EMT and inhibit tumor growth, invasion, and metastasis without causing significant tissue cytotoxicity (221). Similarly, phenyl oxazole derivatives CIB-3b, a regulator of miR-21 biogenesis, interferes with TRBP2 to induce dissociation from Ago2 and Dicer (222).

Many natural products and their derivatives act as novel pathways for cancer therapy by specifically targeting ncRNAs (223). Gomisin M1 is a natural dibenzocyclooctadiene lignan isolated from *Schisandra chinensis*. Its derivatives are thought to be novel TRBP2 modulators that promote the binding of TRBP to Dicer and regulate miR-497-5p, miR-146a-5p, and miR-10b-5p maturation, thereby inhibiting HCC cell proliferation, migration, and invasion (224). Solamargine, a natural alkaloid extracted from Solanaceae plants, downregulates the expression of lncRNA HOTTIP and lncRNA TUG1 and subsequently upregulates the expression of miR-4726-5p that directly targets MUC1. The combination of Solamargine and Sorafenib significantly showed a significantly synergetic effect on MUC1 protein expression, providing a potential strategy for HCC treatment (225). Moreover, Solamargine can induce apoptosis and autophagy in HCC cells through the LIF/miR-192-5p/CYR61/Akt signaling pathway or by stimulating the TIME (226). Sanguinarine is a potent activator of miR-16 expressing wild-type or mutated p53. In Zhang’s research, Sanguinarine activated miR-16-2 expression by increasing p53 occupancy on the miR-16-2 promoter and decreased the expression of miR-16 target genes Bcl-2 and cyclin D1. This effect was validated by anti-miR-16 inhibitor treatment silencing (223).

In addition, we aimed to elucidate the potential mechanisms underlying the anti-cancer properties of various natural active compounds derived from traditional Chinese herbal medicines from the perspective of epigenetic modifications. Notoginsenoside R1 reduces miR-21 expression and subsequently inhibits the PI3K/Akt pathway, thereby exerting anti-HCC activity (227). Oroxin B, a flavonoid monomer compound in the traditional Chinese medicine *Oroxylum indicum* (L.) Vent has a same role (228). Tanshinone IIA may induce hepatoma cell death by downregulating miR-30b transcription and subsequently upregulating PTPN11 levels, which in turn stimulates the SHP2 pathway (229).

In conclusion, RNA-targeted small-molecule modulators have been emerging for almost 20 years, but the FDA has not yet approved miRNA therapies for HCC (230). It is true that miRNAs, which are the pioneers of ncRNAs, still less for lncRNAs and circRNAs. For small-molecule modulators targeting ncRNAs, the feasibility of their therapeutic application in HCC has been proven, but there is still a long way to go before they can become drugs. Their development is facing several hurdles, such as, first, identify specific RNA targets and binding sites and elucidating their mechanisms of action. These challenges all require the development of new design strategies (high-throughput screening, small-molecule microarray screening, structure-based designing, phenotype-based screening, etc.) to screen ncRNAs (216). Second, multi-targeting of endogenous RNAs may be risky for a therapeutic process, and the resulting off-target effects need to be addressed (231). Third, appropriate *in vivo* and *in vitro* models need to be established to confirm the structure-function relationships, potency, and specificity. In conclusion, small-molecule modulators targeting ncRNAs can greatly broaden the range of druggable targets, thus representing a new frontier for drug development. Continued research may lead to breakthrough discoveries while addressing the above-mentioned issues.

## Conclusion

HCC is a multifactorial, multistage malignancy for which it is difficult to obtain satisfactory survival with current treatment options. ncRNAs play an important role in the initiation and progression of HCC and are associated with clinical diagnostic and prognostic properties. This review article focuses on the oncogenic or tumor-suppressive properties of ncRNAs, detailing how ncRNAs regulate key processes (TIME, angiogenesis, EMT, invasion, metastasis, metabolism, and drug resistance) in HCC and their specific mechanisms. Further, we discuss the promising approaches and potential roles of ncRNAs in the field of cancer diagnosis and therapy. Although ncRNAs have shown unique advantages as biomarkers and potential therapeutic targets, they still face challenges. For instance, there remains a vast uncharted territory in ncRNA research, and exploring the emerging role requires advances in next-generation sequencing technologies. Standardized and efficient techniques for rapid and large-scale extraction of ncRNAs have not yet been established. Meanwhile, improved targeting methods and delivery systems are needed to detect and reduce off-target effects. In terms of experimental techniques, off-target effects in knockdown, FISH, and pull-down technologies are difficult to avoid or eliminate completely. Moreover, while the current research on ncRNAs is still carried out at the cellular or animal level, research in clinical settings needs to be advanced to validate key ncRNA functions. Finally, ncRNA-based therapies require interdisciplinary cooperation among various fields, including immunology, molecular biology, pharmacology, and nanotechnology. These findings and challenges reveal the unexpected complexity of ncRNA regulatory mechanisms, which provides many answers but raises more questions. It is believed that, in the near future, ncRNAs can be developed as promising tools for targeted therapies alone or in combination with other therapies.

## Author contributions

QH collected the related paper and drafted the manuscript. MW, XD provided valuable comments. FW, YL and XS supported the funding, designed the manuscript, and revised the manuscript. All authors contributed to the article and approved the submitted version.

## Funding

This study was financially supported by CAMS Innovation Fund for Medical Sciences (CIFMS) (No. 2021-I2M-1-031) and National Key Research and Development Plan Program (2019YFC1710504).

## Acknowledgments

Figures were created with BioRender software (https://biorender.com/ (accessed on 17 September 2022).

## Conflict of interest

The authors declare that the research was conducted in the absence of any commercial or financial relationships that could be construed as a potential conflict of interest.

## Publisher’s note

All claims expressed in this article are solely those of the authors and do not necessarily represent those of their affiliated organizations, or those of the publisher, the editors and the reviewers. Any product that may be evaluated in this article, or claim that may be made by its manufacturer, is not guaranteed or endorsed by the publisher.
